# Harnessing the power of collective intelligence in dentistry: a pilot study in Victoria, Australia

**DOI:** 10.1186/s12903-023-03091-y

**Published:** 2023-06-20

**Authors:** Mahen Ganhewa, Alison Lau, Angela Lay, Min Jae Lee, Weiyu Liang, Emmy Li, Xue Li, Lee Yen Khoo, Su Min Lee, Rodrigo Mariño, Nicola Cirillo

**Affiliations:** 1 CoTreat, CoTreat Pty Ltd, Melbourne, VIC Australia; 2 Melbourne Dental School, The University of Melbourne, 720 Swanston Street, Carlton, VIC 3053 Australia; 3 Center for Research in Epidemiology, Economics and Oral Public Health (CIEESPO), Faculty of Dentistry, Universidad de La Frontera, Temuco, Chile; 4 School of Dentistry, University of Jordan, Amman, 11942 Jordan

**Keywords:** Collective intelligence, Dentistry, Diagnosis, Treatment plan, Wisdom of crowds

## Abstract

**Background:**

In many dental settings, diagnosis and treatment planning is the responsibility of a single clinician, and this process is inevitably influenced by the clinician’s own heuristics and biases. Our aim was to test whether collective intelligence increases the accuracy of individual diagnoses and treatment plans, and whether such systems have potential to improve patient outcomes in a dental setting.

**Methods:**

This pilot project was carried out to assess the feasibility of the protocol and appropriateness of the study design. We used a questionnaire survey and pre-post study design in which dental practitioners were involved in the diagnosis and treatment planning of two simulated cases. Participants were provided the opportunity to amend their original diagnosis/treatment decisions after viewing a consensus report made to simulate a collaborative setting.

**Results:**

Around half (55%, *n* = 17) of the respondents worked in group private practices, however most practitioners (74%, *n* = 23) did not collaborate when planning treatment. Overall, the average practitioners’ self-confidence score in managing different dental disciplines was 7.22 (s.d. 2.20) on a 1–10 scale. Practitioners tended to change their mind after viewing the consensus response, particularly for the complex case compared to the simple case (61.5% vs 38.5%, respectively). Practitioners' confidence ratings were also significantly higher (*p* < 0.05) after viewing the consensus for complex case.

**Conclusion:**

Our pilot study shows that collective intelligence in the form of peers’ opinion can lead to modifications in diagnosis and treatment planning by dentists. Our results lay the foundations for larger scale investigations on whether peer collaboration can improve diagnostic accuracy, treatment planning and, ultimately, oral health outcomes.

**Supplementary Information:**

The online version contains supplementary material available at 10.1186/s12903-023-03091-y.

## Background

Diagnostic error is a detrimental yet common medical error in primary healthcare settings and it is estimated that half of these occurrences have an adverse effect on the long-term outcome of patient centred care [1, 2]. One reason diagnostic errors may occur is that diagnoses of diseases and subsequent treatment planning are often dictated by a single clinician, and such decisions are vulnerable to biases and heuristics of the individual. Judgements made through underlying heuristics are subject to limitations including recalled information, extrapolation, and inaccurate estimations [3]. If this exclusively prevails over critical thought in clinical practice, there may be adverse consequences [4].

Collective intelligence, in contrast to individual aptitude, is the ability of a group to perform a wide variety of tasks with remarkable accuracy [5]. This concept, which can be referred to as “the wisdom of crowds”, is currently used widely in medical settings to help pool expert opinions and boasts superiority as it can aggregate the expertise of individual practitioners. It has been well demonstrated to increase diagnostic accuracy and thus treatment plans [5–8]. It has also been shown that the collective decision making of a group of individuals could outperform even that of a specialist [9]. Collective intelligence thus may play a key role in jolting a clinician out of autopilot, heuristic mode to critical thought whereby patient outcomes are improved.

There are several approaches to harness collective intelligence such as the Delphi workflow, where independent expert inputs are aggregated to form a group consensus. This practice is commonly implemented in the medical field [10–12] and includes, for example, multidisciplinary team meetings and ward rounds. Although the advantage of collective intelligence and its clinical significance is apparent, methods of harnessing collective intelligence have its own limitations. Its effectiveness can be hindered by groupthink, social pressure and conformity bias [13, 14]. Hence, further research into developing collective intelligence methods to encourage contributions in a more structured and independent manner is warranted.

Despite the literature indicating the benefit of collective intelligence in healthcare, dental care is still often dictated by a single clinician. Currently, there is limited research exploring collective intelligence in the dental setting. Hence, this project intends to explore its implementation in dentistry by exploring the impact of a group consensus and whether it changes an individual dentist’s diagnosis, treatment plan and confidence ratings. This is the first study of its kind in Australia, therefore preliminary data gathered from this pilot research may aid guiding future research in dentistry within the realms of harnessing the power of collective intelligence in an effective and efficient manner.

## Methods

This pilot project was carried out to assess the feasibility of the protocol and appropriateness of the study design. The study was undertaken in Victoria, Australia, in accordance with the Declaration of Helsinki principles. It first aimed to investigate the frequency of collaboration amongst dental practitioners and their confidence levels in managing different dental disciplines. Secondly, the project aimed to measure the influence of a group consensus on individual dentists’ treatment plan and confidence ratings.

### Participant recruitment

In essence, sample size calculations are not necessary for pilot studies and hence sample size was not set. This pilot study used a convenience sampling approach. Dental practitioners working in private practice in the state of Victoria were included. Participants were recruited into the study by directly emailing publicly available Victorian dentists and dental practices and by poster advertising distributed on online forums (dental social media groups). As an incentive to participate, the first 30 participants were given a $100 gift voucher if they had completed both stages of the project.

### Survey

The first stage of the study involved an online survey (Qualtrics, Provo, Utah) consisting of 24 questions that assessed general demographic data, workplace, collaboration frequency, and self-confidence ratings ranging from 1 (lowest) to 10 (highest) in managing various dental disciplines (e.g. Periodontics, orthodontics, oral medicine, fixed and removable prosthodontics) and interpreting radiographic modalities [e.g. Cone-beam computed tomography (CBCT) Orthopantomogram (OPG), and bitewings (BW)] (Supplementary Table 1). The questionnaire was validated using a two-step process involving assessment by two experts (one internal (R.M.) and one external to the study) to evaluate whether the questions effectively captured the topic under investigation. Subsequently, the survey was trialled with the members of the Delphi group.

The Delphi group was made up of a panel of 5 dentists (3 males and 2 females, 3 to 15 years’ experience) who were tasked with designing two simulated clinical cases (Case 1 and Case 2) of different complexities. The development of a consensus report was moderated by one of the co-authors (M.G.). Case 1 was designed to be a “complex” case with more treatment considerations involved (Supplementary Table 2), while Case 2 was designed to be a “simple” case (Supplementary Table 3). The cases were identified as simple and complex by the Delphi group based on two categories: the number of evidence based clinical observations present and secondly the intensity of the findings. In the “simple” case, the number of clinical observations and pathologies were agreed to be low (as per group consensus among the Delphi group). The “complex” case had numerous, varied pathologies and clinical observations, with advanced alveolar bone loss being the high impact clinical observation where the Delphi group deemed that intervention was absolutely needed to ensure patient harm prevention.

### Pre-post Intervention diagnostic study

**Fig. 1:**
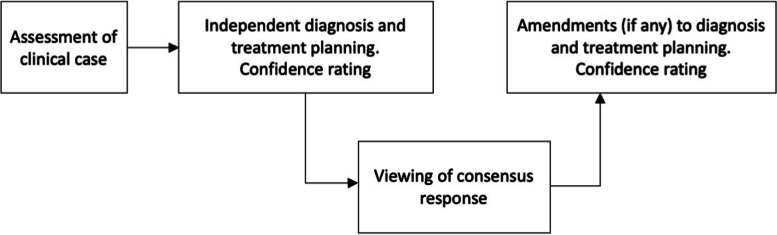
Flowchart illustrating the workflow of the diagnostic study. (1) Assessment of clinical case: participants were provided with a short clinical description and relevant radiographs (2) Independent diagnosis, treatment planning, and confidence rating: participants were asked to anonymously report their individual clinical observations leading to a diagnosis and treatment planning; they also provided a confidence score (1–10) for their decisions (3) Viewing of consensus response: participants were presented with the consensus response which comprised the summary of group discussion from a panel of five dentists (Delphi group) (4) Amendments to diagnosis, treatment planning, and confidence rating: participants were allowed to modify the diagnoses and treatment plans that they made at the beginning, and provided their confidence level for this final decision

Once the participants had finished the online survey, they were invited to complete the second part of the project (Fig. 1), which involved the assessment of the two clinical cases described above. This second stage was a diagnostic study titled “Wisdom of the crowds” and was conducted through an online platform custom built for the study (CoTreat Pty Ltd, Melbourne, Victoria, Australia). By entering the platforms, participants were able to access the two simulated clinical cases (Case 1 and Case 2). The diagnostic study consisted of three main steps (Fig. 1) and would take approximately 20 min for the participants to complete. At the beginning, participants were asked to make preliminary diagnoses and treatment plans individually and anonymously based on the clinical information given including radiographs and relevant symptomatology, presented in the cases. These were open answers and there were no predetermined options from which participants selected diagnoses and treatments. Then, they were asked to rate their diagnosis and treatment planning confidence on a Likert scale from one (No confidence) to ten (Fully confident). When those were completed, the participants were presented with the consensus response which comprised the summary of group discussion from the Delphi group. Following the viewing, participants were allowed to modify the diagnoses and treatment plans that they made at the beginning. Then, the participants were asked to rate their confidence level on a scale from one to ten for the second time. These two sets of ratings obtained from the participants, which were the confidence levels of before and after viewing the consensus response, were evaluated together with their rate of change-of-mind in diagnosis and treatment planning.

Personal details of participants which included their name, emails or phone number were collected to enable researchers to validate solely their identity through the Australian Health Practitioner Regulation Agency (AHPRA) registration database and to contact them for any follow-up.

### Data analysis

Survey responses were exported from Qualtrics. Duplicated and incomplete responses were excluded. The analysis provides basic descriptive information on questions with numeric answers, and mean and standard deviation were calculated with IBM SPSS Statistics for Windows, version 24 (IBM Corp., Armonk, N.Y., USA).

For the intervention study, raw data were exported from the CoTreat diagnostic platform. The diagnostic notes and treatment plans were viewed for every single response. Responses not related to the case were excluded. Based on the diagnostic notes and treatment codes before and after viewing the consensus, researchers annotated whether or not participants had changed their mind. The changes were categorized into change to diagnosis only, change to treatment plan only, and change to both diagnosis and treatment plan. Furthermore, the change in participants’ confidence ratings before and after viewing of consensus were recorded and analysed using one-tailed Wilcoxon’s signed-rank test for both clinical cases. Data were reported as mean (standard deviation). Level of statistical significance was set at *p* < 0.05.

## Results

There were 35 survey responses received, of which 31 were completed and included in subsequent analysis. Two responses were excluded as they were duplicates of existing responses while another two were only partially complete. For the intervention study, a total of 15 responses were received, but only 13 were included in the analysis. Two responses were excluded as the answers were irrelevant to the cases.

### Demographic data

Most of the practitioners (90.32%) who responded were general dentists, while three (9.68%) were dental specialists. The average number of years respondents have been practicing was 15.5 (s.d. 12.15) years (range 2–40), while the average number of days practiced per week was 4.6 (s.d. 0.77) days (range 3–6). The average number of patients seen per day was 10 (s.d. 3.66), range 2–18.

### Work setting and collaboration frequency

Of the 31 respondents, 17 (54.84%) worked in group private practices, 11 (35.48%) worked in solo private practices, two (6.45%) worked in both public and private sectors, and one (3.23%) worked in the public sector only. The majority of practitioners (61.29%) did not work with a dental hygienist or therapists. Most practitioners (74.19%) reported not having their treatment plans audited by a colleague.

On average, survey respondents referred three cases per week to specialists and received an average of two patients per week who were seeking second opinions. For case discussions with colleagues, practitioners discussed an average of two and three cases per week in a formal and casual setting, respectively. Practitioners rarely submitted cases over social media for case discussion, with less than one case on average submitted per week.

### Confidence ratings

**Fig. 2:**
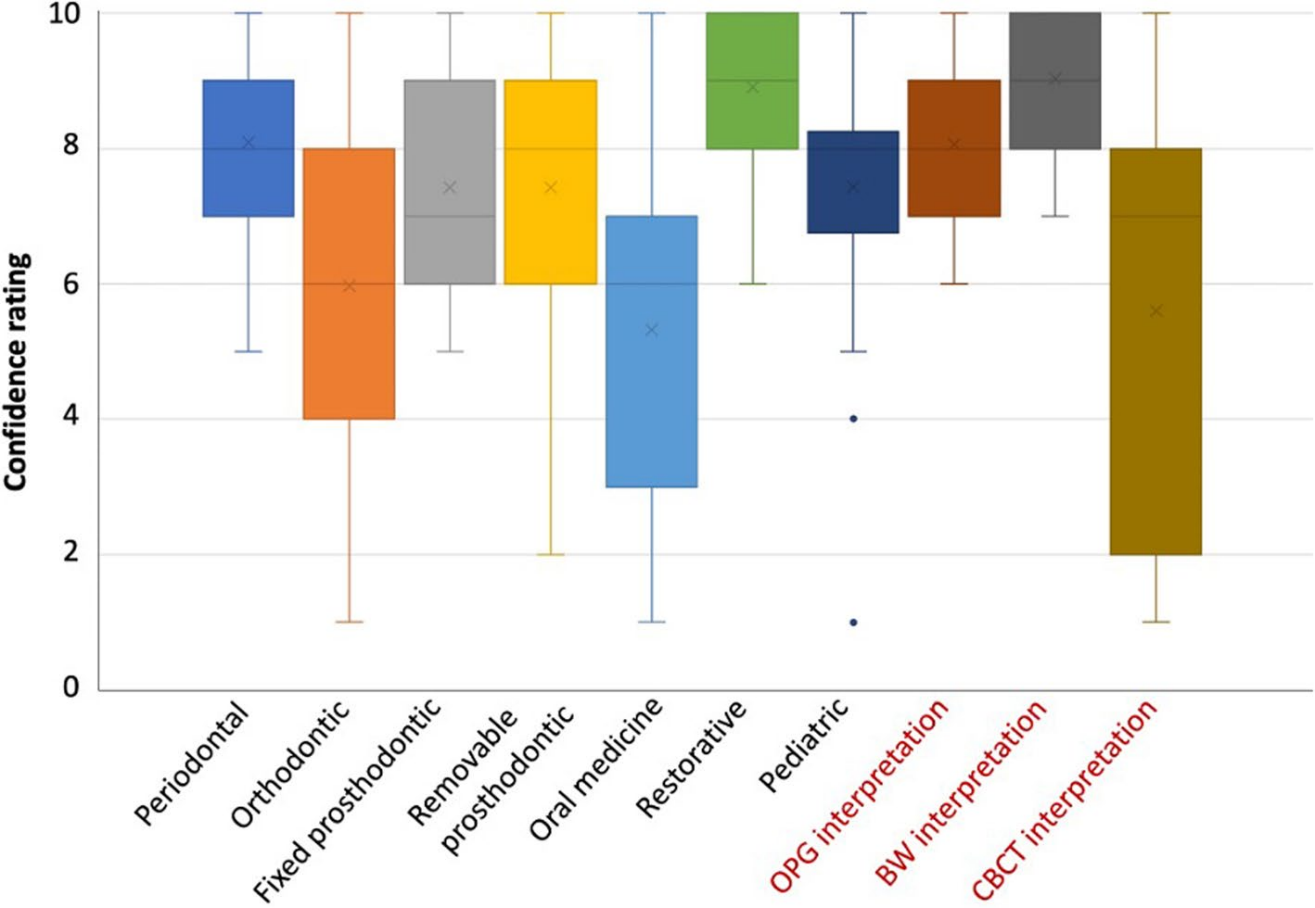
Boxplot showing the confidence ratings of participants on patient concerns across various dental disciplines and types of radiographic interpretation. OPG = orthopantomogram, BW = bitewing, CBCT = cone-beam computed tomography

Overall, mean practitioners’ ratings of their confidence across treating patients of various dental disciplines and interpreting radiographs were 7.22 (s.d. 2.20) and 7.59 (s.d. 2.51), respectively. Practitioners were least confident with oral medicine and orthodontic patient concerns with a mean confidence of 5.32 (s.d. 2.6) and 5.94 (s.d. 2.64) out of 10, both with wide variations (1 to 10). Among radiographic techniques, interpretation of CBCT scored lowest [5.53 (s.d. 3.36)]. Practitioners were most confident with bitewing interpretation and restorative dentistry with mean confidence of 9.03 (s.d. 0.95) and 8.9 (s.d. 1.08) out of 10. Along with periodontal, fixed prosthodontic concerns and OPG interpretation, responses to these five areas had a relatively smaller range with all confidence ratings being at least 5 or higher with the smallest variation being in BW interpretation (range of 7 to 10) (Fig. 2).

The majority of practitioners surveyed (64.52%) were satisfied with their provision of treatment in their daily practice with the rest reporting they were somewhat satisfied. Nevertheless, there was an overwhelming agreement that a more collaborative approach in diagnosis and treatment planning was needed in dentistry, with 30 out of 31 practitioners agreeing.

### Change of mind and confidence rates

For Case 1 (“complex” case), the majority of respondents (*n* = 8; 61.5%) recorded a change in either their initial diagnosis and/or treatment after viewing the consensus. Of those who changed their minds, one changed both their diagnosis and treatment plan; five changed their treatment plans only; and two only changed their diagnosis (Supplementary Fig. 1).

For Case 2 (“simple” case), five responses (38.5%) recorded a change in either their initial diagnosis and/or treatment upon viewing the consensus. The five participants who changed their minds consisted of one who changed both their diagnosis and treatment plan and four who changed their treatment plans (Supplementary Fig. 2).

Overall, based on the data collected for Case 1, the confidence levels of practitioners increased significantly (*p* < 0.05) after viewing the consensus (mean difference 0.87). An increase in the mean confidence level, although not statistically significant, was also present for Case 2 (*p* > 0.05, mean difference 0.51).

## Discussion

In the present study, we provide the first demonstration, albeit preliminary, that collective intelligence has the potential to modify diagnoses and treatment planning in a sample of Victorian dentists. Other healthcare fields have acknowledged the potential benefit of collaboration and have attempted to mobilise it in clinical care [9, 15]. Some commonly documented applications of collaboration are in the management of patients with complex medical conditions in a hospital setting and the rate of implementation of a multidisciplinary team decision [16–19]. The study presented here is a first attempt of applying these concepts to general dental practice in Australia and, most importantly, measuring the impact of collective intelligence in modifying diagnosis and treatment planning. Our results lay the foundations for larger scale investigations on whether peer collaboration can improve diagnostic accuracy, treatment planning and, ultimately, oral health outcomes.

Our research is novel in the sense that no studies to date have examined the application of collective intelligence for diagnosis and treatment planning in a dental setting in Australia. This has salient clinical implications for the profession because the scope of responsibility and demarcation of duties between general practitioners and specialists in dentistry differ from those in medicine [20]. Dentists are in a unique position as they can be regarded as both general practitioners and specialists and, in compliance with their scope of practice, may or may not choose to seek additional input depending on the perceived complexity of the case. Moreover, there may be many treatment modalities for a single presentation which may add to the variance. Advice seeking behaviour among dentists is not widely prevalent. In a study from Canada, the overwhelming proportion of general dentists referred 5 or less patients per week for specialist care [21]. A recent study from Australia shows that only a small percentage (14.7%) of dentists that provide endodontic care were very confident in providing this type of care [22], yet those who were not very confident did not refer their patients to a specialist endodontist. In the survey portion of our study, it was observed that 21% of cases in private dental practices were reviewed collaboratively, either in the form of a specialist referral, second opinion, internet forums, or peer discussion (casual or formal). Referral was the most popular method of collaboration, followed by peer discussions while internet forums were the least popular. Results from a systematic review suggest that dentists prefer to seek the advice of familiar and trusted peers, rather than assessing scientific evidence from literature reviews [23] or posting to a forum of dentists who are of unknown background and training. However, the effectiveness of each collaboration method is not known with respect to improvement in patient management. Therefore, further research is required into what forms of collaboration will be most effective and beneficial to the general dental practitioner and their patients.

The beneficial impact of collaboration seems to be recognised by dentists, with all but one survey respondent in our cohort believing that collective intelligence has a place in dentistry. Currently, the advantages of collaboration have been most clearly demonstrated through increased radiographic diagnostic accuracy [6, 8]. Given that radiography is a mainstay of dentistry, it is imperative that all dentists should be comfortable with interpreting diagnostic imaging. Our survey results indicated that clinicians were most confident with interpreting 2D radiographs with small field of view (e.g. bitewing radiographs) compared with 3D diagnostic imaging. Improving a clinician’s certainty in interpreting radiographs by inviting peers to review the same image may improve treatment outcomes. In a meta-analysis by Kurvers et al., the confidence of the clinician’s diagnosis was correlated with increased levels of sensitivity and specificity [24]. Although assessment of diagnostic accuracy was outside the scope of this study, the effect of collective intelligence on the relationship between confidence and diagnostic accuracy should be further investigated in a dental context.

The second part of the study sought to explore the effect of collective intelligence in diagnosis and treatment planning. It was observed that participants were more prone to changing their treatment plans when given a “complex” case involving multiple diagnoses and treatment elements. It was observed that more dentists changed their own treatment plans after viewing treatment plans of their peers to include more elements that they previously did not consider. Changes to diagnoses and treatment planning, especially with complex cases, was likely the result of having access to a wider range of options that that were not previously available. Therefore, collaboration with peers may lead to more thorough treatment planning by dentists. This is particularly relevant if we consider that huge variations exist in clinical decision-making in dentistry [25], including orthodontics [26], restorative dentistry [27] and periodontics [28]. These discrepancies may be due to the fact that while clinical decision-making is a complex process, individual clinical judgements are often made intuitively on limited heuristics to simplify decision making. A recent scoping review identified six major recurring themes that influence dental practitioners' clinical decision-making: clinical factors, clinical experience, patient preferences and perceptions, heuristics and biases, artificial intelligence and informatics, and existing guidelines [29]. With the application of collective intelligence, not only are dentists more thorough with their treatment plans, but they are also exposed to a wider range of treatment with less outlying treatments. As a result, the patient benefits from a holistic and thoroughly considered treatment. Moreover, our findings suggests that dentists may feel more reassured after receiving additional input from other dentists.

For cases perceived as “simple”, the influence of collective intelligence may be diminished. This may be because the approach for management of simpler cases has fewer acceptable options, and dentists are generally more confident with their treatment plan even before viewing the consensus. Nonetheless, even in “simple” cases, variation in the treatment plans was noted. Furthermore, in cases where dentists’ treatment plans were different to the consensus, some dentists kept their original plan (e.g., to restore certain lesions instead of monitoring and remineralisation that was suggested by the consensus). In summary, although the influence of collective intelligence was less prominent in “simple” cases, the effect is not negligible, with more than one third of participants changing their minds when presented with the consensus treatment. Hence, collective intelligence may have a potentially beneficial role in dentistry to improve clinical outcomes.

The main limitations of our research study relate to the design and implementation of the intervention study. Firstly, the consensus report was created by a panel of five external dentists moderated by one of the co-authors (M.G.). While the size of this Delphi group may be regarded as small, published studies in health applications have used panel sizes from as low as 4 individuals, bearing in mind that size “should be governed by the purpose of the investigation” [30]. Also, collective intelligence benefits from anonymous decision-making, but the consensus treatment plan was obtained via open discussion. This research study design was necessary to simplify the research workflow. It is worth noting that in our pilot study, “collective intelligence” was rendered by the Delphi group, not by the pooled responses of individual dentists. However, it is possible that collective responses from dentists can be taken as a model of collective intelligence in future studies. Secondly, as the study was completed during the 2021 pandemic, there was difficulty in engaging with dentists across Victoria, Australia. A small sample size of 13 participants completed both parts of the study, and some questions may have yielded different results due to the reduced number of patients that could be seen during lockdowns. Although it was not the main outcome of this pilot trial, the sample size may not have had enough power to detect changes. As such, the results from our study cannot be readily generalizable to real-life clinical settings. Besides that, the limitations of recall inaccuracies may have also affected results of collaboration frequencies. Thus, any results are not definitive of any outcome (either positive or negative). As the diagnostic study was a simulated clinical setting, the Hawthorne effect may also have predisposed some participants to provide a diagnosis and treatment plan that may not have been as thorough in a private setting. Additionally, conformity bias may be in play, with individual practitioners deciding to readily change their diagnosis and treatment plan to suit the consensus report especially as they are aware that they are being part of a research study. This may mean that data collection may not be representative of reasoning undertaken in the dental clinic. Interestingly, a statistically significant increase in confidence levels seen in Case 1 (complex case) of the diagnostic study suggests that the consensus treatment plan may add to the clinicians’ confidence that they are providing a standard of care on par with their peers. However, it must be noted that participants who did not change their mind were not given a chance to record their confidence level after viewing the consensus. As only matched pairs could be analysed, the resulting sample size was even smaller. Finally, while patient outcomes could be improved, feasibility (time) and viability (costs) remain challenges to implementing collective intelligence to daily practice. Despite these limitations, this pilot study clearly points towards a potential usefulness of collective intelligence and group diagnosis in dental care.

## Conclusion

Our study has provided insight into the extent of collaboration in dentistry, with limited collaborative effort currently being employed in dental diagnosis and treatment planning. Our pilot study shows promising results that collective intelligence could potentially lead to a more thorough diagnosis and treatment planning by dentists, resulting in better patient care and treatment outcome. Moreover, it may also potentially provide reassurance to practitioners’ confidence in diagnosis and treatment planning. Larger studies with more clinicians from different backgrounds, diagnosing cases of different dental disciplines and of varying complexities anonymously are warranted, to allow for extrapolation of the effects of collective intelligence in allowing for better clinical outcomes for different cases. Additionally, more studies into the methods of implementation of collective intelligence in the dental clinic should also be explored if we are to improve clinical outcomes for patients via collective intelligence.

## Supplementary Information

**Additional file 1: Table S1.** Survey Questions. **Table S2.** Case 1 of the diagnostic study with case scenario and consensus responses from five dentists with varying clinical experience. **Table S3.** Case 2 of the diagnostic study with case scenario and consensus responses from five dentists with varying clinical experience. **Figure S1.** Bar chart illustrating the different aspects where participants changed their mind for Case 1. **Figure S2.** Bar chart illustrating the different aspects where participants changed their mind for Case 2.

## Data Availability

The data that support the findings of this study are available from the corresponding author upon reasonable request.
