# Supplementary material for: Harnessing the power of collective intelligence in dentistry: a pilot study in Victoria, Australia
Source: BMC Oral Health. 2023 Jun 20;23:405. doi: 10.1186/s12903-023-03091-y (PMC10280903; doi:10.1186/s12903-023-03091-y)

## **Supplementary Material**

**Table S1**

| **Survey Questions** |
| --- |
| Q1. How many years have you been a practicing dentist? |
| Q2. Do you have specialist training? - Selected Choice |
| Q3. How many days per week do you typically practice? |
| Q4. What sort of practice? - Selected Choice |
| Q5. How many patients on average would you see per day? |
| Q6. In your last FULL working week, how many patients did you refer to a specialist? |
| Q7. In your last FULL working week, how many cases did you discuss formally with a peer or peer group? |
| Q8. In your last FULL working week, how many cases did you casually discuss with a peer? (over lunch etc) |
| Q9. In your last FULL working week, how many cases did you post on a facebook group (or similar) to receive additional opinions from peers? |
| Q10. In your last FULL working week, how many patients did you see that were seeking a second opinion from you? |
| Q11. Do you have a dental hygienist or therapist that works alongside you? |
| Q12. Does a superior or employer occasionally audit or supervise your treatment plans? |
| Q13. How confident are you when DIAGNOSING and treatment planning a patient with periodontal concerns? |
| Q14. How confident are you when DIAGNOSING and treatment planning a patient with orthodontic concerns? |
| Q15. How confident are you when DIAGNOSING and treatment planning a patient with fixed prosthodontic concerns? |
| Q16. How confident are you when DIAGNOSING and treatment planning a patient with removable prosthodontic concerns? |
| Q17. How confident are you when DIAGNOSING and treatment planning a patient with oral medicine concerns? |
| Q18. How confident are you when DIAGNOSING and treatment planning a patient with restorative concerns? |
| Q19. How confident are you when DIAGNOSING and treatment planning for a paediatric patient? |
| Q20. How confident are you when interpreting an OPG? |
| Q21. How confident are you when interpreting a posterior bitewing? |
| Q22. How confident are you when interpreting CBCT? |
| Q23. In relation to the treatment plans you have implemented for your own patients, how would you rate satisfaction level? |
| Q24. Do you believe that dentistry requires a more collaborative approach in regards to diagnosis and treatment planning? |

*Table S2.* Case 1 of the diagnostic study with case scenario and consensus responses from five dentists with varying clinical experience.

| **Case 1** | |
| --- | --- |
| *Case scenario* | A 33 year-old female patient with no significant medical history presents to you with persistent pain from tooth 38. The patient has not been to a dentist for several years. The 38 becomes symptomatic every few months and abates but presently the pain is persistent and the patient cannot control her pain with paracetamol and ibuprofen.    Upon examination, limited jaw opening was noted along with minor extra oral facial swelling and lymphadenopathy on the LHS.    The patient is motivated for optimal dental treatment, and wants your recommendation about a treatment plan. Please create a provisional treatment plan (using item codes and notes) based on the scenario provided and your findings in the image provided. Include any initial and ongoing care if relevant. |
| *Radiograph* | 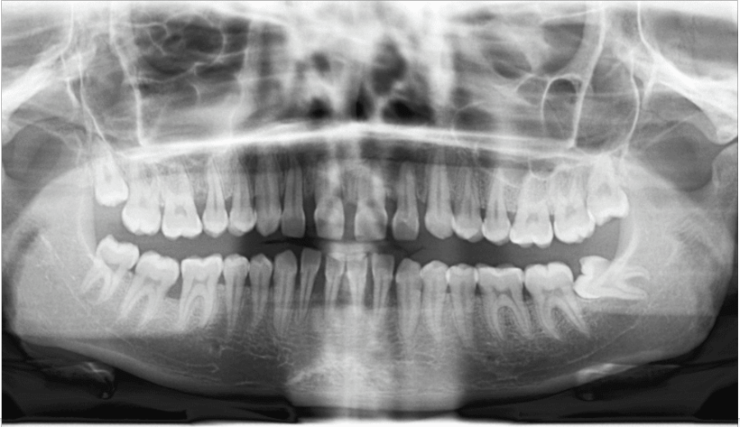^(21)^ |
| *Diagnosis Note* | · Pericoronitis tooth 38  · Generalised periodontitis  · Treatment:  o Provide analgesic, antibiotics. Refer to OMFS for management of all 8s  · Discussions:  o Discuss risks of facial swelling, pericoronitis.  o Discuss risks of wisdom teeth extractions.  o Discuss generalised periodontitis.  o Discuss comprehensive examination + periodontal chart needed.  o Discuss referral to periodontist. |
| *Treatments* | · 927 Provision of medication/medicament  · 019 Letter of referral  · 013 Oral examination – limited    · 221 Clinical Periodontal analysis and recording  · 022 Intraoral X-ray  · 022 Intraoral X-ray  · 011 Comprehensive oral examination  · 019 Letter of referral |

*Table S3.* Case 2 of the diagnostic study with case scenario and consensus responses from five dentists with varying clinical experience.

| **Case 2** | |
| --- | --- |
| *Case scenario* | A 41 year old male patient presents to you for a routine check-up and clean. The patient has recently moved from interstate, and cannot see his regular dentist whom he usually sees 6 monthly. The patient has no relevant medical or dental history. His dental restorations were placed approximately 10 years ago. BPE/PSR reveals the following:    2 \| 1 \| 2  2 \| 2 \| 2    The patient is motivated for optimal dental treatment, and wants your recommendation for a treatment plan. Please create a provisional treatment plan based on the scenario and images provided. Including any initial and ongoing care if relevant. |
| *Radiograph* | 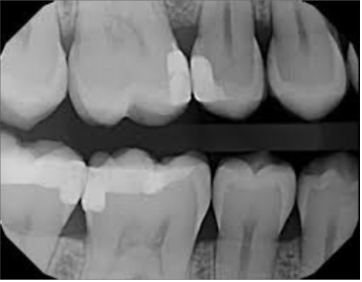^(22)^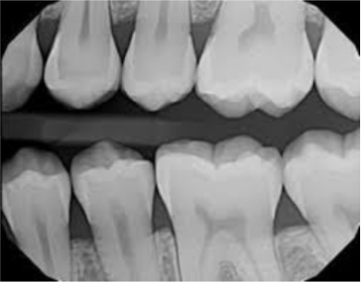^(23)^ |
| *Diagnosis Note* | · Diagnosis  o Watch teeth 14, 15, 24 for incipient caries.  · Treatment:  o Routine scale and polish, Topical fluoride (Duraphat) for incipient lesions. No further treatment required.  · Discussions:  o Discuss oral hygiene and workshop technique if needed.  o Discuss diet.  o Discuss importance for 6 monthly cleans and 12-24 monthly PBWs. |
| *Treatments* | · 012 Periodic oral examination  · 114 Removal of calculus – first appointment  · 121 Topical application of remineralisation and/or cariostatic agents, one treatment |

**Legend to Supplementary Tables**

**Table S1**: Survey questions.

**Table S2:** Case 1 of the diagnostic study with case scenario and consensus responses from five dentists with varying clinical experience.

**Table S3:** Case 2 of the diagnostic study with case scenario and consensus responses from five dentists with varying clinical experience.

**Supplementary Figures**

**Figure S1**: Bar chart illustrating the different aspects where participants changed their mind for Case 1.


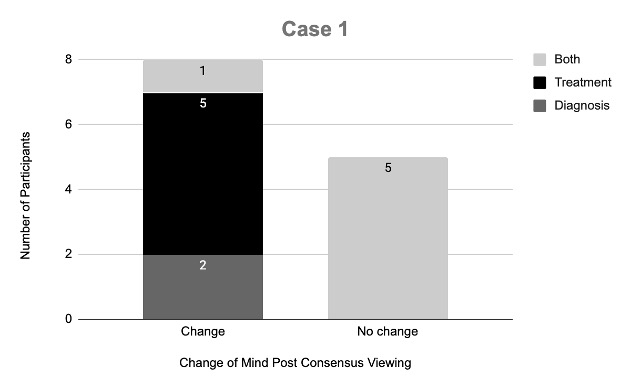


**Figure S2**: Bar chart illustrating the different aspects where participants changed their mind for Case 2.


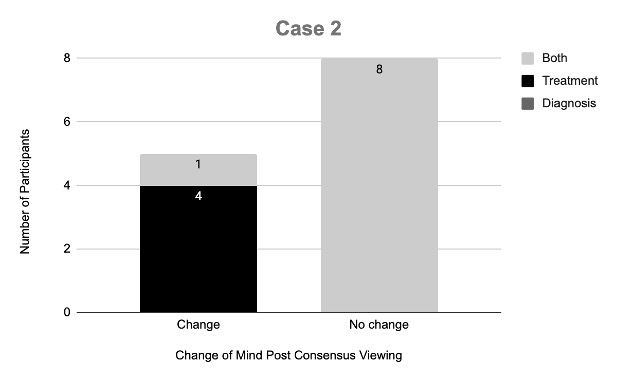

Supplement: Supplementary file 1 — Additional file 1: Table S1. Survey Questions. Table S2. Case 1 of the diagnostic study with case scenario and consensus responses from five dentists with varying clinical experience. Table S3. Case 2 of the diagnostic study with case scenario and consensus responses from five dentists with varying clinical experience. Figure S1. Bar chart illustrating the different aspects where participants changed their mind for Case 1. Figure S2. Bar chart illustrating the different aspects where participants changed their mind for Case 2. [file 12903_2023_3091_MOESM1_ESM.zip › Supplementary Material.docx]
