# Supplementary figures and images for: Harnessing the power of collective intelligence in dentistry: a pilot study in Victoria, Australia
Source: BMC Oral Health. 2023 Jun 20;23:405. doi: 10.1186/s12903-023-03091-y (PMC10280903; doi:10.1186/s12903-023-03091-y)

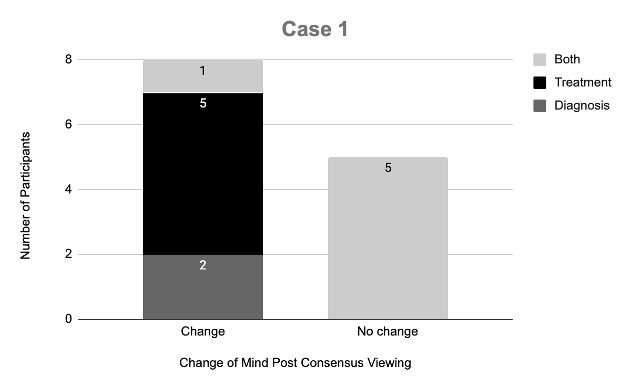

Supplement: Supplementary file 1 — Additional file 1: Table S1. Survey Questions. Table S2. Case 1 of the diagnostic study with case scenario and consensus responses from five dentists with varying clinical experience. Table S3. Case 2 of the diagnostic study with case scenario and consensus responses from five dentists with varying clinical experience. Figure S1. Bar chart illustrating the different aspects where participants changed their mind for Case 1. Figure S2. Bar chart illustrating the different aspects where participants changed their mind for Case 2. [file 12903_2023_3091_MOESM1_ESM.zip › Figure S1.jpg]

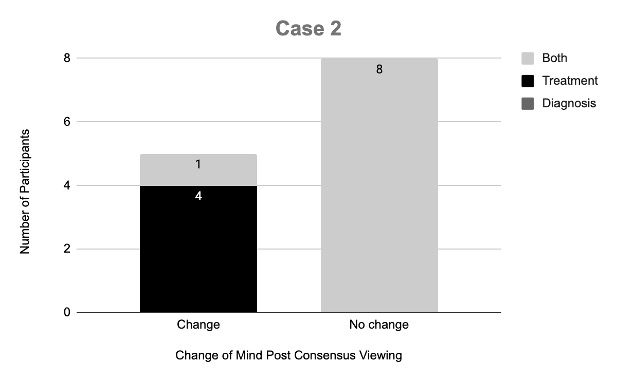

Supplement: Supplementary file 1 — Additional file 1: Table S1. Survey Questions. Table S2. Case 1 of the diagnostic study with case scenario and consensus responses from five dentists with varying clinical experience. Table S3. Case 2 of the diagnostic study with case scenario and consensus responses from five dentists with varying clinical experience. Figure S1. Bar chart illustrating the different aspects where participants changed their mind for Case 1. Figure S2. Bar chart illustrating the different aspects where participants changed their mind for Case 2. [file 12903_2023_3091_MOESM1_ESM.zip › Figure S2.jpg]
